# Accurately Predicting Protein p*K*_a_ Values Using Nonequilibrium Alchemy

**DOI:** 10.1021/acs.jctc.3c00721

**Published:** 2023-10-11

**Authors:** Carter J. Wilson, Mikko Karttunen, Bert L. de Groot, Vytautas Gapsys

**Affiliations:** † Department of Mathematics, The University of Western Ontario, N6A 5B7 London, Canada; ‡ Centre for Advanced Materials and Biomaterials Research (CAMBR), The University of Western Ontario, N6A 5B7 London, Canada; § Department of Physics & Astronomy, The University of Western Ontario, N6A 5B7 London, Canada; ∥ Department of Chemistry, The University of Western Ontario, N6A 5B7 London, Canada; ⊥ Computational Biomolecular Dynamics Group, Department of Theoretical and Computational Biophysics, Max Planck Institute for Multidisciplinary Sciences, 37077 Göttingen, Germany; # Computational Chemistry, Janssen Research & Development, Janssen Pharmaceutica N. V., Turnhoutseweg 30, B-2340 Beerse, Belgium

## Abstract

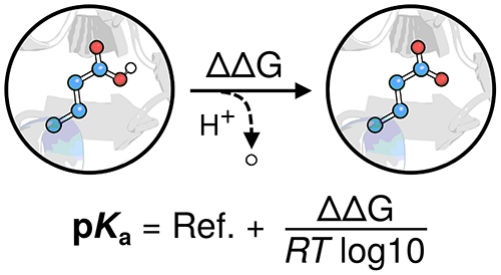

The stability, solubility, and function of a protein depend on both its net charge and the protonation states of its individual residues. p*K*_a_ is a measure of the tendency for a given residue to (de)protonate at a specific pH. Although p*K*_a_ values can be resolved experimentally, theory and computation provide a compelling alternative. To this end, we assess the applicability of a nonequilibrium (NEQ) alchemical free energy method to the problem of p*K*_a_ prediction. On a data set of 144 residues that span 13 proteins, we report an average unsigned error of 0.77 ± 0.09, 0.69 ± 0.09, and 0.52 ± 0.04 p*K* for aspartate, glutamate, and lysine, respectively. This is comparable to current state-of-the-art predictors and the accuracy recently reached using free energy perturbation methods (e.g., FEP+). Moreover, we demonstrate that our open-source, pmx-based approach can accurately resolve the p*K*_a_ values of coupled residues and observe a substantial performance disparity associated with the lysine partial charges in Amber14SB/Amber99SB*-ILDN, for which an underused fix already exists.

## Introduction

Amino acids with ionizable side chains make up approximately 30% of the residues found in proteins^1,2^ and play a key role in maintaining protein stability,^3−6^ modulating solubility,^7,8^ mediating protein–protein interactions,^9,10^ and facilitating cell signaling.^11,12^ These amino acids, namely, aspartate, glutamate, arginine, lysine, cysteine, tyrosine, and histidine, are functionally dependent on their protonation states, which vary depending on their local environments. The measure of this dependence is known as the p*K*_a_, which relates the pH of the solution to the protonation state of a residue via the Henderson–Hasselbalch equation, i.e., p*K*_a_ = pH + log[HA]/[A^–^]. Given its degree of solvent exposure, Coulombic interactions, and hydrogen bonding, the p*K*_a_ of an amino acid residue may be raised or lowered relative to its reference p*K*_a_^°^—determined using a capped peptide (e.g., ACE-AXA-NH_2_) in solution—resulting in a lower or higher likelihood of protonation at a given pH. For acidic groups, the p*K*_a_ values tend to be elevated relative to their reference,^13−15^ while for basic groups, the p*K*_a_ values tend to be lowered relative to their reference.^16,17^ These shifts away from the reference value can reach up to ±5 p*K* units, and in many proteins, key ionizable residues are situated in such a way that a perturbation of their p*K*_a_ allows them to perform unique and specific functions.^18−22^ The existence of such functional motifs relies on the alterable stability of the covalent bond between hydrogen and its heavy atom (e.g., O–H and N–H). The tendency of a side chain containing these groups to (de)protonate in a given microenvironment is quantified by the p*K*_a_.

The relationship of protein–ligand binding to p*K*_a_ is of particular interest.^23−25^ Here, the p*K*_a_s of both the ligand and the binding site residues as well as the pH- and binding-induced conformational changes of the protein are all intimately related. Resolving the precise states of the ionizable residues, as well as the local conformations of the apo and holo protein, are active fields of study that involve both experimental^26−28^ and computational approaches.^29−32^

The conventional and often most precise method to determine the p*K*_a_ of an ionizable side chain is to measure the pH dependence of the main or side-chain chemical shifts using multidimensional nuclear magnetic resonance (NMR) spectroscopy.^33−35^ The dependence of the chemical shift on pH is then fit to the Henderson–Hasselbalch equation, and the p*K*_a_ is resolved from the point of inflection. NMR can estimate the p*K*_a_ with an accuracy of 0.1–0.2 p*K* unit;^36^ however, this strongly depends on the nuclei considered (i.e., ^13^C vs ^15^N) and the fit to the Henderson–Hasselbalch curve, which can be difficult due to conformational changes,^37^ titration coupling,^38^ or if the chemical shift simply reports a different titration event.^36,39^ Even with the above caveats, NMR remains the experimental method of choice to resolve p*K*_a_ values in proteins and is, in general, very reliable. There are alternative approaches for measuring p*K*_a_ values including fluorometry, kinetic assays, and isothermal titration calorimetry.^40−42^ However, they have their own challenges and generally obtain p*K*_a_ values with higher uncertainty compared to the NMR-based approach.

**Figure 1:**
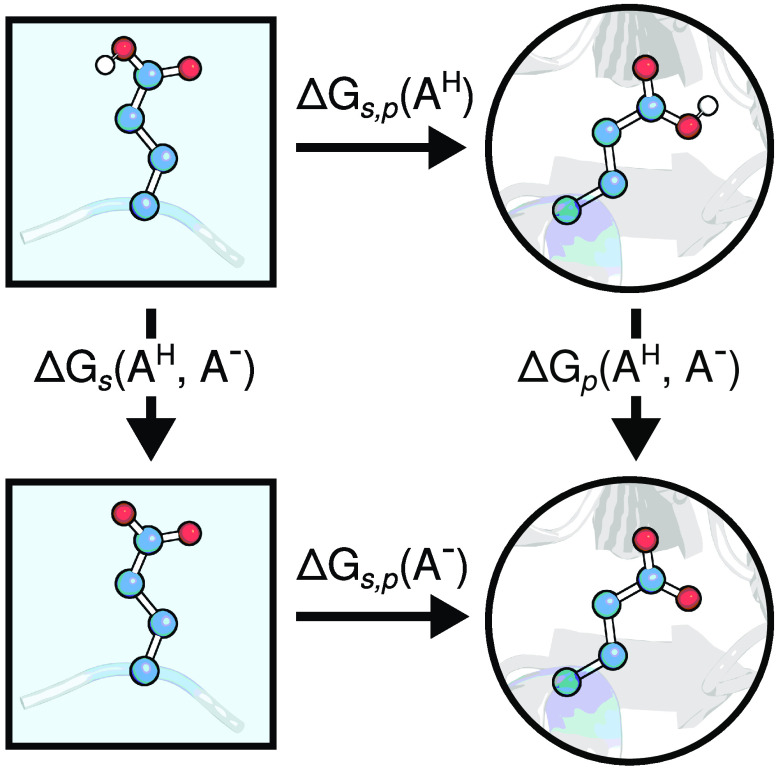
Thermodynamic cycle to compute the free energy difference between protonating a residue in a capped peptide in solution and the same residue in a protein. This ΔΔ*G* can be related to p*K*_a_(protein) given the reference p*K*_a_^°^ via eq 1.

Theoretical methods are a compelling alternative to experiments. Many of these are motivated by a free energy formalism based on the thermodynamic cycle shown in Figure 1. Here, we consider a residue of interest (*A*) in both protein (Figure 1, right) and reference peptide in solution (Figure 1, left). We assume that the reference p*K*_a_^°^ is known, then p*K*_a_(protein) is given by
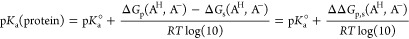
1Note that ΔΔ*G*_p,s_(A^H^, A^–^) implicitly contains two terms. The first (ΔΔ*G*^env^) represents the free energy of dissociating a proton within a protein relative to the reference state (e.g., capped peptide), where the protein residues are fixed to some state such that the value is pH independent; the second (Δ*G*^titr^(pH)) accounts for the contribution from the titratable residues in the protein as they (de)protonate with pH. A consideration of the first component yields a , which can be further used to calculate the true p*K*_a_
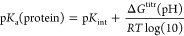
2In most cases, it is safe to assume that the mutual dependence (or coupling) of a residue A and its protein microenvironment is small, and by simply assigning residues to the charge state most likely for a corresponding model compound in solution (e.g., capped peptide) at pH ≈ 7.4, we can assume p*K*_a_(protein) ≈ p*K*_int_. However, there are cases where this assumption will fail, and a consideration of Δ*G*^titr^(pH), at least for nearby titratable residues, is necessary to correctly resolve p*K*_a_(protein). To that end, we have elsewhere introduced a thermodynamic-cycle-based formalism to account for this additional titration contribution and therein discuss the role of microscopic p*K*_a_ values in the context of coupled residues.^43^

Whether or not the pH dependence on the p*K*_a_ is taken into account, the fundamental aim of most theoretical methods is to resolve the free energy difference in eq 1 and thus estimate the p*K*_a_. This can be done within a macroscopic or microscopic framework; we briefly describe both.

Macroscopic frameworks model the entire system, protein, and solvent as either a regularly shaped or an irregularly shaped object situated within a dielectric medium. From this, the energy terms can be resolved using the Poisson–Boltzmann equation (PBE). For a regularly shaped protein (e.g., idealized sphere), the PBE can be solved analytically;^44,45^ however, for a more realistic, irregularly shaped protein, the PBE must be solved numerically. The numerical Poisson–Boltzmann (PB) approach for computing p*K*_a_ values was pioneered by Bashford and Karplus^46^ and has since been continually refined.^47^ Changes in both the underlying algorithmic and numerical formalism (e.g., parameter selection, linearized PBE,^48^ etc.) and the structural descriptions of the system (e.g., partial charge changes,^49^ side-chain rotamers,^50^ etc.) have aimed to increase accuracy and applicability.

A microscopic framework based on atomistic simulations,^51^ unlike a macroscopic one, in theory, does not require the definition of empirical parameters (e.g., charge density) or physical quantities (e.g., permittivity). The principal drawback is the computational cost that can be overcome by modification of the underlying model representation or implementation (e.g., the reintroduction of pseudoparameters) or by improvements in computing power. Molecular dynamics (MD) simulations offer an attractive solution for sampling biomolecular ensembles spanning meaningfully long time scales with fully atomistic representations of both protein and solvent. These simulations and the resultant ensembles might be used as an input for a PB-based approach,^52−55^ or can be performed in conjunction with a free energy method (e.g., thermodynamic integration,^56,57^ free energy perturbation,^58^ etc.), allowing for a direct resolution of the ΔΔ*G* between protonation states. An alternative MD-based approach is constant pH molecular dynamics (CpHMD) simulations. Here, Monte Carlo sampling^59−62^ (discrete CpHMD) or λ-dynamics^63−66^ (continuous CpHMD) is used to explicitly sample protonation events. This allows for an explicit consideration of the proton concentration, where the protonation states of titratable residues are not restrained but are allowed to dynamically follow the free energy gradient.

Empirical (EM) approaches stand in contrast to those described above, which are primarily based on a rigorous free energy formalism. Empirical methods tend to rely on sets of approximate functional forms (e.g., hydrogen bonds) with knowledge-based parameters that are optimized based on large training sets of measured p*K*_a_ values. Such approaches have generated predictors with impressive accuracy at low computational cost,^67,68^ which have been further enhanced with the advent of machine learning.^69,70^

It can be said that for all of the methods mentioned above, the objective is to provide predictive accuracy within the same range as that reached by experiment (i.e., <0.2 p*K* units). A perfect method ought to be system independent and hence not require fitting to experimental data. It should be able to robustly predict the free energies of protonation in the core of a protein and in the solvent-exposed regions, which requires that solute–solvent interactions be accurately represented. Moreover, the ability to change environmental conditions (e.g., temperature and ionic concentration) is another necessary requirement.

Alchemical free energy calculations based on molecular dynamics (MD) simulations have the potential to fulfill these requirements. Previous work has demonstrated that nonequilibrium (NEQ) free energy methods are able to accurately estimate the effects of mutations on protein stability,^71^ as well as relative^72^ and absolute protein–ligand binding affinities.^73^ However, the ability to seamlessly and consistently extend these free energy frameworks to pH-dependent contexts, where invariably differences in the residue protonation states will measurably shift the computed free energies, and where assignment of the protonation states requires knowledge of the p*K*_a_ values, first requires a successful demonstration that plain p*K*_a_ values can be resolved using NEQ.

To this end, we use pmx-based NEQ free energy calculations to compute the ΔΔ*G* and corresponding p*K*_a_ values (as described in eq 1) for 144 residues across 13 different proteins in two contemporary force fields. The calculated free energy differences were combined into a consensus estimate. We also consider six popular and well-validated alternative computational methods as a comparison. Additionally, we compare our results to p*K*_a_ values computed using FEP+^58^ (Schrödinger Inc.) and observe no statistically significant difference between the accuracy achieved with both methods. We also report substantial performance disparities for lysine residues in Amber14SB,^74^ which are caused by the partial charge assignment of the backbone and for which corrections already exist.^75^ Furthermore, we demonstrate the ability of our pmx-based approach to accurately resolve the pH-dependent p*K*_a_ values of coupled residues, expanding the potential use for probing amino acids involved in unique redox or catalysis reactions. The average unsigned error (AUE) of the pmx-computed p*K*_a_ values across the residue classes considered was 0.68 ± 0.05 p*K*. The open-source pmx tool^76^ is freely available at https://github.com/deGrootLab/pmx.

## Methodology

### Data Sets

The structures for the p*K*_a_ calculations were taken from the PDB database. Identifiers (and the corresponding experimental p*K*_a_ data) are as follows: 1BPI(77) (data^78,79^), 1BNR(80) (data^81^), 1BEO(82) (data^83^), 6QFS(84) (data^84^), 3BDC(85) (data^85^), 1CLB(86) (data^87,88^), 1RGG(89) (data^90^), 2LZT(91) (data^36^), 4TRX(92) (data^93^), 2RN2(94) (data^95^), 1OMU(96) (data^97^), 1NZP(98) (data^99^), and 1LKJ(100) (data^18^) (see the Supporting Information (SI) for details about 1LKJ). The list of proteins, their residues, and the corresponding experimental p*K*_a_ values are provided in Table S1.

PDB structure IDs for thermostability calculations and the corresponding experimental ΔΔ*G* data references are as follows: 1EY0(101) (data^17,102^), 2LZM(103) (data^104−110^), and 2RN2(94) (data^111^). The list of proteins, their residues, and the corresponding experimental ΔΔ*G* values are provided in Table S2.

We make reference to four main p*K*_a_ data sets:

- full: 13 proteins and 144 residues: 57 aspartate, 48 glutamate, and 39 lysine residues (main data set used for method comparison; all other data sets are subsets)
- FEP+: contains the 65 residues that overlap with a recent FEP+ publication^58^ (used to compare NEQ and FEP+ approaches)
- lysine: contains 13 lysine residues from hen egg-white lysozyme (HEWL) and calbindin 9k (used to assess the source of a lysine performance discrepancy)
- reduced: contains 15 aspartate and 14 glutamate residues from SNase + ΔPHS and HEWL (used to assess Amber99SB-*disp* performance)

### Nonequilibrium Alchemy

pmx(76) was used for the system setup, hybrid structure and topology generation, and analysis. Initial structures were taken from the PDB database (see the Methodology section).

A double system in a single box setup was used; here, both the protein and peptide (e.g., ACE-AXA-NH_2_) are situated at a distance of 3 nm in the same box, which ensures charge neutrality during the alchemical transition.^112^ To prevent consequential protein–peptide interactions, a single Cα in each molecule was positionally restrained. Given the thermodynamic cycle used (Figure 1), the free energy cost associated with this restraint cancels between the two vertical branches. We used the CHARMM36m^113^ (with CHARMM-modified TIP3P^114^) and Amber14sb^74^ (with TIP3P^115^) force fields.

For all systems, an initial minimization was performed by using the steepest descent algorithm. A constant temperature corresponding to the reference experimental setup was maintained implicitly using the leapfrog stochastic dynamics integrator^116^ with an inverse friction constant of γ = 0.5 ps^–1^. Pressure was maintained at 1 bar using the Parrinello–Rahman barostat^117^ with a coupling time constant of 5 ps. The simulation time step was set to 2 fs. Long-range electrostatic interactions were calculated using the particle-mesh Ewald method^118^ with a real-space cutoff of 1.2 nm and a Fourier spacing of 0.12 nm. Lennard-Jones interactions were force-switched off between 1.0 and 1.2 nm. Bonds to hydrogen atoms were constrained using the Parallel LINear Constraint Solver.^119^

To improve sampling, systems were run for 25 ns in four independent replicas; in each case, the first 5 ns were discarded as equilibration. From the remaining 20 ns, 200 nonequilibrium transitions of 200 ps were generated and work values from the forward and backward transitions were collected using thermodynamic integration. These values were then used to estimate the corresponding free energy with Bennett’s acceptance ratio^120^ as a maximum likelihood estimator relying on the Crooks fluctuation theorem.^121^ Bootstrapping was used to estimate the uncertainties of the free energy estimates,^112,122^ and these were propagated when calculating the ΔΔ*G* values. By varying the length of equilibrium and transition simulations as well as the number of transitions, we ensured that this simulation protocol yields converged free energy estimates (Figure S1). Equation 1 was used to convert between ΔΔ*G* and p*K*_a_(protein) values using the corresponding references (i.e., aspartate: 3.94 ± 0.03, glutamate: 4.25 ± 0.05, and lysine: 10.4 ± 0.08).^123,124^

### Conventional Predictors

In addition to the MD-based p*K*_a_ estimation, we also considered an empirical (EM) method PropKa^67,68^ (v3.4); four Poisson–Boltzmann (PB) methods: DelPhiPKa^125,126^ (v2.3), H++^127^ (v4.0), MCCE^50,128^ (v2.8), and PypKa^129^ (v2.9.4); and evaluated a machine-learning-based predictor p*K*_a_-ANI^70^ (v.0.1.0).

PropKa is an empirical predictor, where the Δ*G* contributions are captured by Coulombic, desolvation, and intrinsic electrostatic (e.g., hydrogen bonding) energy equations. Default settings were used when performing the calculations.

DelPhiPKa, as with all PB methods considered here, calculates the electrostatic potential by numerically solving the PBE using a finite difference method. Based on DelPhi software, this method uses a smooth Gaussian function to capture the heterogeneous dielectrics of the solute and solvent. Default settings were used except for the salt concentration, which was set according to the experimental setup (Table S1).

H++ relies on the single-conformer version of MEAD^130^ and assigns charges and parameters based on Amber99SB. Default settings were used except for the default pH, which was set to 7.4, and the salt concentration, which was set according to the experimental setup (Table S1).

MCCE, based on DelPhi, uses Monte Carlo simulations to capture dynamic side-chain conformational changes. Default settings were used except for the salt concentration, which was set according to the experimental setup (Table S1).

PypKa uses Monte Carlo calculations to probe the proton tautomers and employs DelPhi to solve the PBE. Default settings were used, except for the salt concentration, which was set according to the experimental setup (Table S1).

p*K*_a_-ANI can also be considered an empirical predictor. This predictor utilizes deep representation learning^131^ that combines an atomic environment vector and the neural network potential ANI-2x.^132^ Default settings were used when performing the calculations, including a gas-phase minimization of the initial PDB structures in GROMACS using the Amber14SB force field.

## Results

### Overall Performance

Double free energy differences (ΔΔ*G*) were calculated for all 144 residues (48 aspartates, 57 glutamates, and 39 lysines), allowing us to robustly evaluate performance on a large data set. For the MD-based and PB-based approaches, a consensus estimate was used to make comparison easier. The EM-based approach corresponds to PropKa calculations, while the ML-based p*K*_a_-ANI method is discussed in a separate section.

With respect to the MD approach, we observed two important sources of prediction inaccuracy: residue coupling and lysine parametrization. Adjusting the p*K*_a_ calculation framework to account for these led to an adjusted estimate that we compare to the unadjusted one. This is extensively discussed in the Determinants of Accuracy: Lysine Parametrization and Determinants of Accuracy: Protonation Neighborhood and Residue Coupling sections.

**Figure 2:**
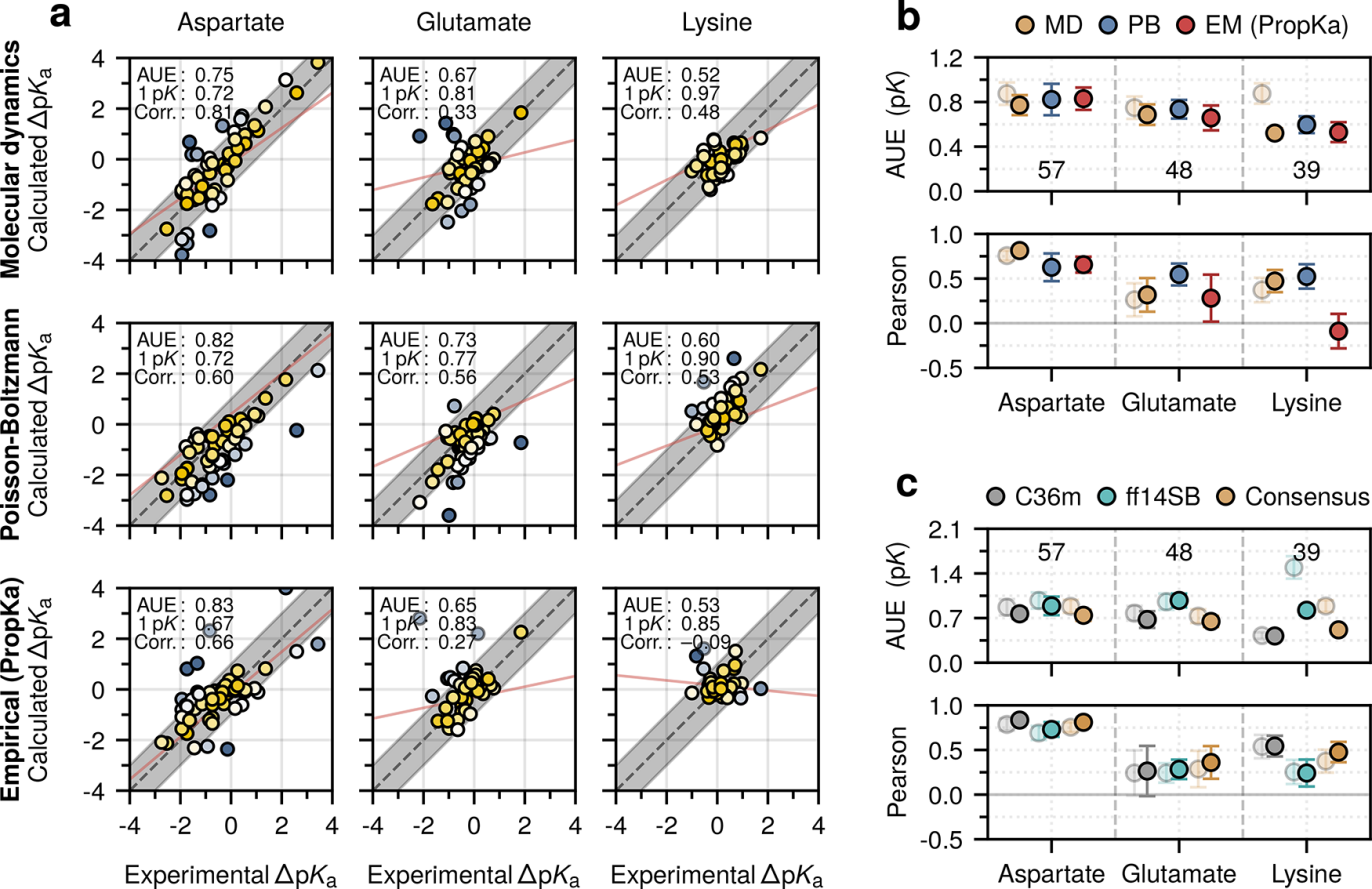
Full data set residue-wise performance. (a) Correlation between the calculated and experimental p*K*_a_ values. MD values are adjusted for residue coupling and lysine parametrization. Marker color indicates deviation from experiment. Regression lines are indicated in red. The proportion of residue 1 p*K* units from experiment is indicated. (b) Average unsigned errors (AUEs) and Pearson correlation coefficients computed for the various methods: molecular dynamics (MD), Poisson–Boltzmann (PB), and the empirical PropKa approach (EM). (c) AUEs and Pearson correlation coefficients were computed for the two force fields: CHARMM36m and Amber14SB, and their consensus. Transparent markers indicate the unadjusted estimates. Numerical values indicate the number of residues considered. When available, bootstrapped standard errors are depicted.

Figure 2 summarizes the main findings: in absolute terms, MD-based nonequilibrium free energy calculations perform comparably to conventional in silico predictors, with an overall adjusted predictive AUE of 0.68 ± 0.05 p*K* taken as an average over each residue class (compared to 0.74 ± 0.07 and 0.70 ± 0.06 p*K* for the consensus of the Poisson–Boltzmann (PB) methods and empirical (EM) PropKa method, respectively) (Figure 2a,b). Regarding the individual residue classes computed using the MD approach, for the adjusted estimate, AUEs were 0.77 ± 0.09 p*K* (aspartate), 0.69 ± 0.09 p*K* (glutamate), and 0.52 ± 0.04 p*K* (lysine) (Figure 2a,b).

The unadjusted force-field differences revealed that CHARMM36m performed as well or better for each residue class compared to Amber14SB (Figure 2c). The most notable differences were evident for lysine, where Amber14SB significantly underperformed compared to CHARMM36m (AUE: 0.42 ± 0.05 vs 1.48 ± 0.18 p*K*).

**Figure 3:**
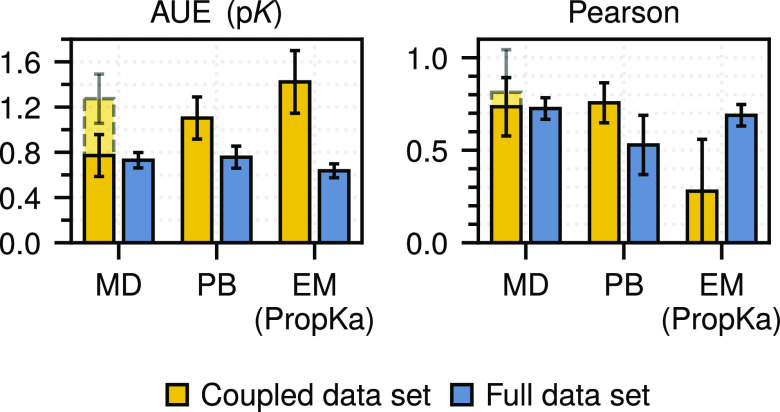
Performance of methods on coupled residues. Average unsigned errors (AUEs) and Pearson correlation coefficients were computed for both the coupled data set (i.e., 18 aspartates and glutamates) and the full data set aspartate and glutamate residues, with the coupled set discarded. Dashed lines indicate the performance of the MD-based approach before coupling was accounted for (see text). Bootstrapped standard errors are depicted.

The Pearson correlation coefficients revealed a similar trend; for aspartate and lysine, the adjusted MD-based estimate gave values of 0.81 ± 0.04 and 0.48 ± 0.12, respectively, performing as well or better than the alternative approaches (PB: 0.61 ± 0.16 and 0.52 ± 0.13; EM (PropKa): 0.67 ± 0.08 and −0.09 ± 0.19). For glutamate, weaker correlations with the MD-based approach (0.33 ± 0.19) were evident. Regardless of the method, the highest correlations were for aspartate, where the experimental p*K*_a_ values had the largest dynamic range, while the weakest correlations were for lysine, where the dynamic range of the experimental values was narrower (Figure 3).

We did not observe a strong dependence of the prediction accuracy on the protein system. Rather, the systems for which higher accuracy was observed (Figure S2) contained a higher proportion of probed lysine residues (e.g., 1NZP and 1LKJ), again illustrating disparate p*K*_a_ prediction accuracy for different residue types. In general, residues with larger Δp*K*_a_ values (Figure S3) and lower solvent exposure (Figure S4) tended to be predicted worse. We note that these two variables are related: probed residues with smaller Δp*K*_a_s were also found to be more solvated (Figure S5).

### Determinants of Accuracy: Lysine Parametrization

**Figure 4:**
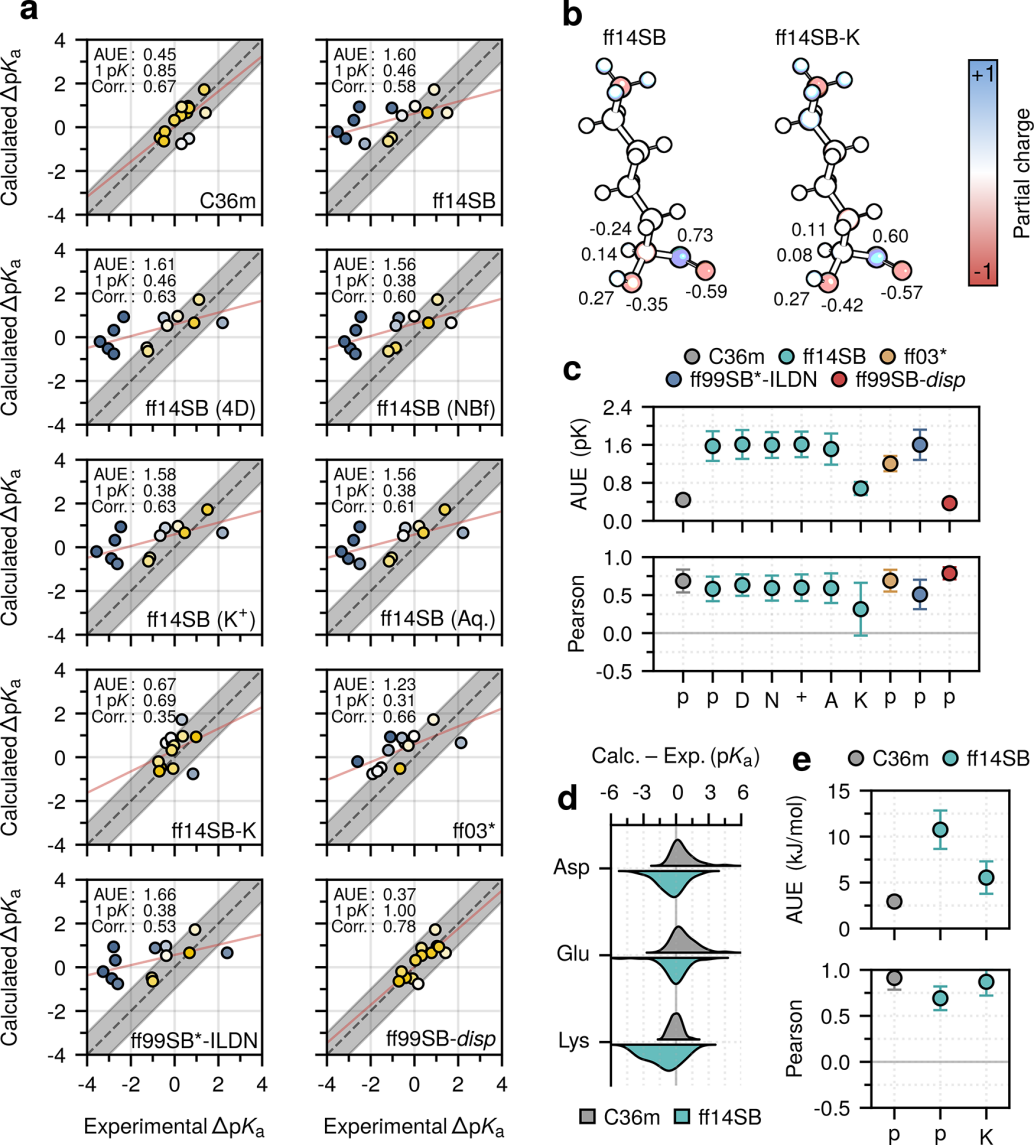
Calculating lysine p*K*_a_ values with different force fields. (a) Correlation between the calculated and experimental p*K*_a_ values. Marker color indicates deviation from experiment. Regression lines are indicated in red. The proportion of residues 1 p*K* unit from experiment is indicated. (b) Partial charge assignment differences between Amber14SB and Amber14SB-K. Numeric values correspond to backbone atoms. (c) Average unsigned errors (AUEs) and Pearson correlation coefficients computed for the various force-field combinations: five variants of Amber14SB (with TIP4P-D (D), with NBFIX (N), with K^+^ counterions (+), with Åqvist ions (A), or with Best et al. charges assigned to the probed lysine (K)), as well as “plain” (p) CHARMM36m, Amber14SB, Amber03*, Amber99SB*-ILDN, and Amber99SB-*disp*. (d) Distribution of differences between the unadjusted MD-based and experimental p*K*_a_ values. (e) AUEs and Pearson correlations computed on a lysine thermostability data set. When available, bootstrapped standard errors are depicted.

As discussed above, Amber14SB provided markedly poorer estimates of the ΔΔ*G* compared with CHARMM36m for most of the lysine residues considered, significantly underestimating the p*K*_a_ values (Figure 4a,d). We conceived of two potential sources of error: (1) environmental and (2) residue parametrization. Given the discussions in the literature pertaining to ion overbinding^133−135^ and the role of a solvent model on protein solvation,^136^ we began by assessing the role of environmental conditions. Specifically, we probed K^+^ (rather than Na^+^) counterions, NBFIX parameters,^134^ Åqvist^137^ (rather than Joung/Cheatham^138^) ion parameters, and TIP4P-D water^139^ (rather than TIP3P). Using these variants, the p*K*_a_ values of lysines from a 13 residue data set (i.e., hen egg-white lysozyme (HEWL) and calbindin 9k) were computed. No significant improvement in the estimates was observed (Figure 4a,c).

To consider the role of parametrization, simulations were performed with three different versions of Amber, namely, Amber99SB*-ILDN,^140−142^ Amber03*,^141,143^ and Amber99SB-*disp*.^144^ On the same lysine data set, a dramatic improvement was observed with Amber99SB-*disp* (Figure 4a,c). Given that differences in the dihedral parametrization between Amber99SB*-ILDN and Amber14SB appeared to confer almost no performance improvement, this narrowed the likely cause of the difference to the nonbonded interactions. Regarding the Lennard-Jones terms, Amber99SB-*disp* alters the parameters of aspartate, glutamate, and arginine, leaving open the possibility of more accurate interactions between lysine and other charged residues in the protein as the source of this discrepancy. However, more notable was the inclusion of the Best et al. lysine partial charges (i.e., Amber99SB*-ILDN-Q^75^) with Amber99SB-*disp*. Although both Amber14SB and Amber99SB*-ILDN have the same partial charge assignment, Amber99SB-*disp* uses altered backbone charges for aspartate, glutamate, lysine, arginine, and doubly protonated histidine (Figure 4b). These were originally developed in the Amber99SB*-ILDN-Q force field to correct for aberrant helical propensities and create consistency among the amino acids. In both Amber99SB*-ILDN and Amber14SB, with the exception of proline, all but these five charged residues have the same assigned backbone partial charge set for C, O, N, and HN. By using the updated parameters by Best et al., both protonated (LYS) and deprotonated (LYN) lysine in Amber99SB*-ILDN-Q and Amber99SB-*disp* have the same charge assignment for C, O, N, and HN.

Such a backbone partial charge assignment is akin to that in the CHARMM36m force field, which has the same backbone partial charge sets (including the Cα and Hα atoms) for all residues except proline and glycine.

We constructed a hybrid Amber14SB-K force field with the altered lysine partial charges but only for the probed residue. We found that this force field performed markedly better on the lysine data set, cutting the average unsigned error by almost half, from 1.48 ± 0.18 to 0.81 ± 0.08 p*K* (Figure 4a,c). The improvement was most pronounced for lysine residues in the helical regions (Figure S6). This result, in addition to that from Best et al.,^75^ suggested that the default partial charges of lysine were erroneous. To further assess the effect of partial charges, we computed the thermostability of 15 lysine mutations using CHARMM36m, Amber14SB, and Amber14SB-K. We again observed a marked improvement in the AUE using the altered lysine partial charges, which shifted the value from 10.42 to 5.54 kJ/mol (Figure 4e).

While Amber99SB-*disp* exhibited the highest accuracy on the lysine data set (Figure 4c), suggesting its general use for p*K*_a_ prediction, this behavior did not hold for aspartate and glutamate. On a reduced data set (i.e., SNase + ΔPHS and HEWL), Amber99SB-*disp* exhibited below-average accuracy (Figure S7).

### Determinants of Accuracy: Protonation Neighborhood and Residue Coupling

Overall, alchemical free energy calculations and conventional p*K*_a_ predictors provide comparable accuracy. However, unlike many alternative approaches, the alchemical method described here allows for the resolution of conditional p*K*_a_ values. The consideration of such values may not only improve the estimates but also allow one to determine the pH-dependent p*K*_a_ of a residue. Recently, we derived a formalism to conveniently combine double free energy differences from alchemical calculations in order to account for coupling between residues when predicting the p*K*_a_.^43^

In this work, we selected 18 residues, including several acidic dyads across the data set, for which the deviation from experiment was >1 p*K*. We further calculated the p*K*_a_ values of these residues by taking into account possible couplings with the protonatable residues in their neighborhood. For residues neighboring a histidine, standard p*K*_a_ calculations were performed in the presence of doubly protonated histidine, i.e., we assume this to be the protonation state at the pH where aspartate and glutamate titrate. For pairs of nearby (i.e., <0.5 nm) acidic residues, we applied the aforementioned thermodynamic formalism, while for apparent triads, an assessment of the most probable deprotonation event was first determined, followed by an application of the formalism on the remaining dyad. Explicitly accounting for residue coupling reduced the AUE from 1.28 to 0.76 p*K* of the residues considered, bringing the accuracy close to the AUE observed over the full data set (Figure 3). For all of the methods considered, this coupled residue subset had higher errors than those observed on the remaining aspartate and glutamate residues (i.e., full data set minus coupled subset).

We note that this analysis was retrospective, where we have a priori access to the correct p*K*_a_ values, i.e., we could preselect which residues to subject to these more involved calculations involving inter-residue couplings. However, in principle, such calculations can be applied to any residues with nearby protonatable neighbors. Our formalism^43^ ensures that if alchemical calculations suggest no coupling, the final p*K*_a_ estimate will remain similar to that of a standard calculation without coupling considerations.

### Method Comparison

**Figure 5:**
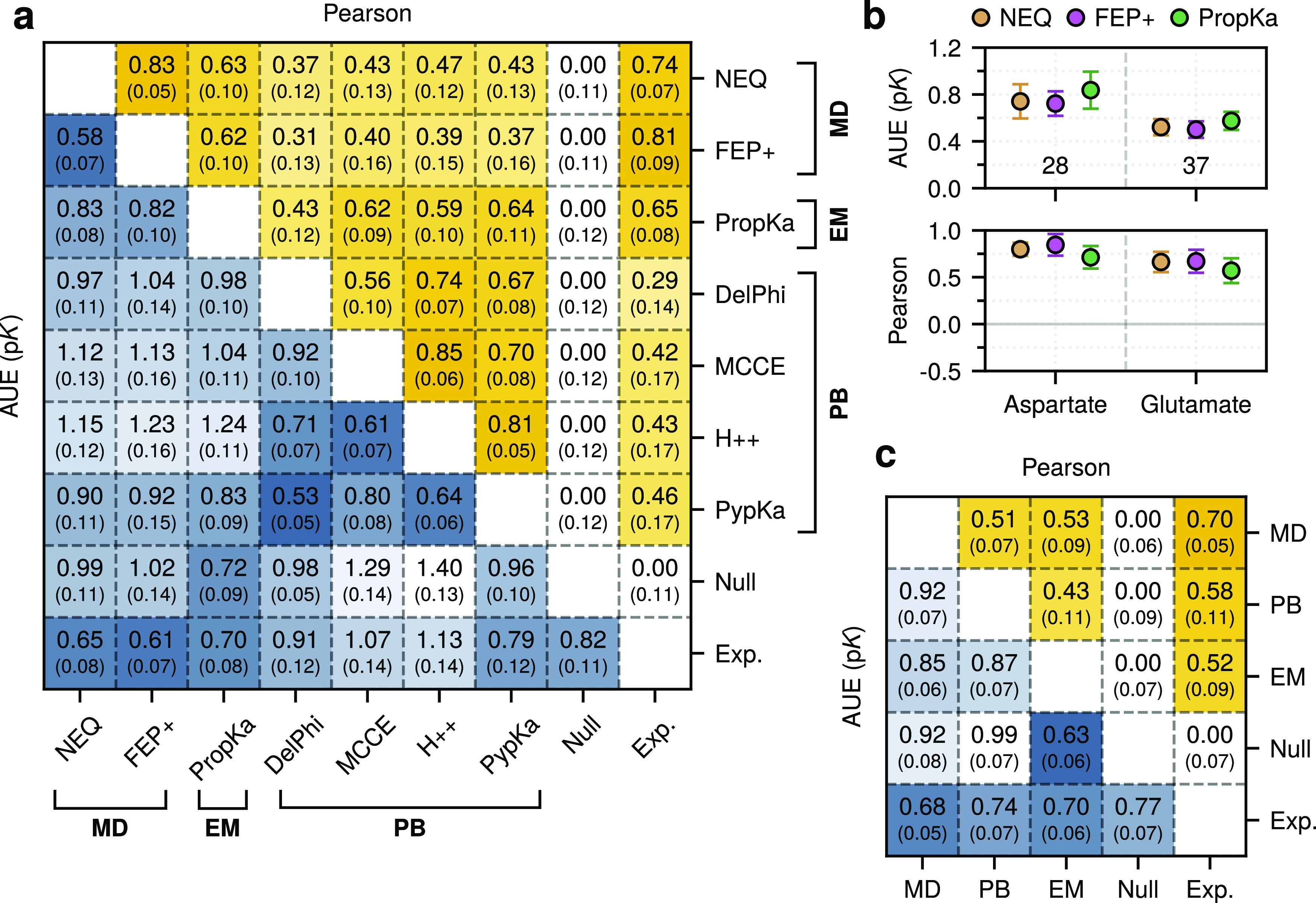
Comparison of the Δp*K*_a_ predictions by each method. (a) Pearson correlations (upper right triangle) and AUEs (lower left triangle) between Δp*K*_a_ estimates were calculated for each method over the FEP+ data set. Comparison with experiment means that the bottom row and rightmost column correspond to the overall performance. DelPhiPKa is abbreviated DelPhi. (b) Individual residue-wise error plot of the NEQ, FEP+, and PropKa methods on the FEP+ data set. Numerical values (i.e., 28 and 37) indicate the number of residues considered. (c) Pearson correlations and AUEs for the three Δp*K*_a_ consensus estimates were calculated over the full data set; note that EM corresponds to PropKa. Comparison with experiment means that the bottom row and rightmost column correspond to overall performance. Bootstrapped standard errors are indicated.

Recently, FEP+ was used to compute the p*K*_a_ values of 79 aspartate and glutamate residues.^58^ We observed comparable performance on the overlapping 65 residue data set (referred to as the FEP+ data set); the average unsigned error was 0.65 ± 0.08 for NEQ and 0.61 ± 0.07 for FEP+ (Figure 5a), and the Pearson correlation coefficients were 0.74 ± 0.06 and 0.80 ± 0.09, respectively. These represented the two strongest performing methods on the FEP+ data set. We also assessed the degree of correlation between the Δp*K*_a_ estimates for both methods; here, the Pearson correlation coefficient was 0.83 ± 0.05, suggesting a strong relationship (Figure 5a). This was the second strongest correlation between any two methods on the FEP+ data set. Regarding residues, glutamate p*K*_a_ values were predicted with a higher accuracy than aspartate (Figure 5b).

We also considered our NEQ approach in relation to individual computational methodologies (rather than a consensus), including the popular PropKa software. Given the computational efficiency of this empirical method, it presents a compelling approach for large-scale p*K*_a_ calculations. We found that NEQ and FEP+ could outperform PropKa on the FEP+ data set (Figure 5a,b); however, PropKa still showed strong performance on the full data set (Figures S8 and S9). For the full data set, while the AUE values for PropKa predictions were small, the correlations also tended to be weaker. This was particularly evident for lysine, where the Pearson correlation coefficient was near zero. For the precise discrimination of individual residues and an absolute ordering of p*K*_a_ values, an MD-based free energy approach may be warranted.

As with FEP+, we evaluated the degree of correlation and deviation between the Δp*K*_a_ values computed using various methods. The strongest correlations were observed within method classes (e.g., DelPhiPKa/MCCE) rather than between them (e.g., DelPhiPKa/NEQ). Strong correlations were particularly evident within the PB-based approaches when evaluating on both the FEP+ data set and the full data set (Figures 5a and S9).

Probing the full data set revealed a general decrease in the AUE and stronger correlations with experiment (Figure S9). Given that the FEP+ data set contains a higher proportion of glutamates to aspartates and no lysines, this result suggests that data set composition can impact performance and should warrant consideration in future benchmarks.

Both MD-based methods, NEQ and FEP+, showed high levels of agreement with each other and with experiment. The rather weak intermethod correlation is further emphasized by comparing consensus results from the method families over the full data set (Figure 5c).

Comparison with a null model revealed stronger correlations over the FEP+ and full data sets for all methods considered (Figures 5a and S9). However, the average unsigned errors for several approaches were not significantly different from the errors of the null model. The MD-based approach exhibited consistent performance even for residues with |Δp*K*_a_| > 1 (Figure S3), performing significantly better than the null model, where the AUE degrades linearly with Δp*K*_a_.

Overall the MD-based approach was the only method to match or significantly exceed the null model with respect to the average unsigned error and Pearson correlation coefficient across all three residue classes (Figure S10). Among the predictors, both PropKa and PypKa performed well on the FEP+ and full data sets; with the exception of p*K*_a_-ANI, these represent the two strongest performing, non-MD methods evaluated here.

### Machine Learning Predictor p*K*_a_-ANI

We also evaluated the performance of a promising, recently developed machine-llearning-based predictor, p*K*_a_-ANI. Unfortunately, the set of p*K*_a_ values collected in this work largely overlapped with the training set of p*K*_a_-ANI. As the evaluation of an ML approach on its training set should not be used to judge the accuracy of the method, we present this evaluation only for the sake of completeness (Figure S8). As expected, p*K*_a_-ANI performance on the full data set was strong, exceeding the other methods with respect to AUE (0.44 ± 0.07 p*K*) and Pearson correlation coefficient (0.87 ± 0.05).

**Figure 6:**
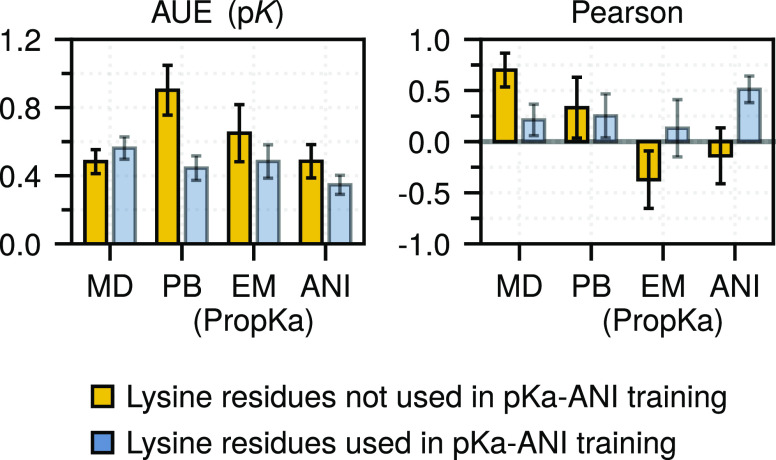
Performance of methods on the lysine subset (14 values), which were not in the p*K*_a_-ANI training set. The performance on the rest of the lysine set (25 values) is shown as a reference. Bootstrapped standard errors are depicted.

To gain a more realistic insight into the performance of p*K*_a_-ANI, we considered a small subset of 14 p*K*_a_ values from the full data set that did not appear in the training set of p*K*_a_-ANI. This set, however, contains only lysine residues from two protein systems. The observed accuracy on this subset was 0.49 ± 0.10 p*K* with a correlation of −0.18 ± 0.27 (compared to 0.48 ± 0.07 and 0.71 ± 0.16 with the MD-based approach) (Figure 6).

We can assess the accuracy difference between the “train” and “test” sets by evaluating the performance of p*K*_a_-ANI on a lysine p*K*_a_ subset that was used to train the predictor.

While in terms of AUE the performance of the ML-based predictor becomes only insignificantly worse, the reduction in the Pearson correlation coefficient between the “test” subset and the “training” set is significant. Given the small size of the “test” data set and bias toward only one residue type, this evaluation of p*K*_a_-ANI accuracy should not be overinterpreted. Nevertheless, our analysis suggests a reduction in prediction accuracy when using independent test data, a result consistent with the original p*K*_a_-ANI publication.^70^

## Discussion

Here, we assess the ability of NEQ-based free energy calculations to resolve the p*K*_a_ values of 144 residues across 13 proteins. Although large-scale studies on the application of NEQ alchemical calculations for predicting mutagenic folding free energy changes and relative and absolute ligand-binding affinities already exist, such an extension to protein p*K*_a_ values has been absent from the literature. A seamless free energy workflow that can probe the role of protonation on ligand binding, particularly relevant at an enzymatic active site, and resolve the underlying p*K*_a_ values of both individual residues and bound molecules is highly desirable. Here, we take a step toward that goal. Although (de)protonation is the smallest topological change that a residue can undergo, it results in a significant charge shift. We find that such perturbations and the corresponding free energies can be readily resolved using our pmx-based approach (i.e., AUE: 0.68 ± 0.05 p*K*), with accuracy comparable to FEP+,^58^ and demonstrate the ability of this approach to resolve the p*K*_a_ of coupled residues. While the MD-based approach can capture protein dynamics and account for residue coupling, with both contributing to the accurate p*K*_a_ predictions, it is a computationally expensive method. Based on the timings from the current work, running simulations for 1 week on a single GPU (RTX 2080 Ti) would allow for computing 12 p*K*_a_ differences in an average-sized protein domain (≈100 residues).

Our results reveal that the Amber14SB^74^/Amber99SB*-ILDN^142^ partial charges for lysine are likely erroneous, yielding p*K*_a_ and thermostability estimates that deviate significantly from experiment. Importantly, we demonstrate that this error can be resolved using charges assigned in Amber99SB*-ILDN-Q.^75^ Taken alongside those by Best et al., our results do suggest that the Amber14SB backbone partial charges warrant further investigation; however, we do not advocate the use of Amber14SB-K until further validation is performed. One interesting point of investigation could be determining whether these modified charges resolve previously documented ion-overbinding problems^135^ and conformational discrepancies in polyelectrolytes.^145^

While our results and the recent work of others^58,146^ underscore the p*K*_a_ prediction accuracy attainable by MD-based free energy methods, the gap between prediction and experiment remains larger than the experimental error of 0.1–0.2 p*K* units.^36^ In the current work, we have identified two main sources contributing to the p*K*_a_ prediction error: residue coupling and force-field parametrization.

With respect to the first, we have demonstrated that accounting for the coupling of nearby titratable sites plays a crucial role in accurate p*K*_a_ prediction. While this requires additional calculations within the alchemical free energy framework,^43^ it brings a significant improvement to the prediction accuracy (Figure 3).

Regarding the second, we found that the deprotonated lysine backbone partial charges in Amber14SB are more favorable relative to the protonated backbone charges, which, in turn, results in a p*K*_a_ underestimation. In support of this hypothesis was the observation that the effect was largest for residues situated in regions where backbone interactions are most prominent (e.g., α-helix). Our finding underscores the importance of accurately parametrizing both the protonated and deprotonated forms of the amino acids and the sensitivity that relative free energy calculations can have to seemingly minor parametrization differences. Suggestive of this phenomenon was the recent demonstration^147^ that modification of the Amber14SB cysteine thiolate parameters—to agree more closely with ab initio solvation data—could improve the p*K*_a_ prediction accuracy by 0.5 p*K* units when combined with an MD-based approach.^146^ The use of polarizable force fields might also improve p*K*_a_ estimates;^148^ however, recent work using Monte Carlo simulations with the Drude force field and a Poisson–Boltzmann continuum solvent model did not show a significantly improved prediction accuracy.^149^

We note that conformational sampling may also play a role; however, this is less significant in the systems probed here. For proteins with more pronounced pH-dependent conformation shifts, local rearrangements over tens of nanoseconds may be insufficient to capture the end-state distributions and would result in poorer estimates of the p*K*_a_.^150,151^

In summary, we have shown that our open-source, pmx-based NEQ free energy method performs on par with state-of-the-art commercial software and achieves an average unsigned error that meets or exceeds alternative in silico predictors when assessed on independent test data. Furthermore, this MD-based approach yielded markedly stronger correlations with experiment, suggesting better performance for the discrimination of residues with similar p*K*_a_s. Additionally, our observation of a significant partial charge discrepancy suggests that high-quality experimental p*K*_a_ values may constitute a compelling data set to be used during force-field parametrization.

## Data Availability

PDB structures, simulation setup files, and calculated p*K*_a_ values are available at https://github.com/deGrootLab/pka_prediction_2023.

## Supporting Information Available

Assessment of the simulation protocol (Figure S1); protein-wise AUEs and Pearson correlation coefficients (Figure S2); AUE for individual methods is shown as a function of Δp*K*_a_ (Figure S3); AUE for the two force fields used as a function of solvation number (Figure S4); solvation number as a function of Δp*K*_a_ interval (Figure S5); AUEs comparison between Amber14SB and Amber14SB-K for specific secondary structure elements (Figure S6); residue-wise AUEs and Pearson correlation coefficients computed on an aspartate/glutamate reduced subset for Amber99SB-*disp* (Figure S7); residue-wise AUEs and Pearson correlation coefficients for individual methods (Figure S8); Pearson correlation coefficients and deviations (AUEs) of the Δp*K*_a_ between methods (Figure S9); residue-wise *p*-value analysis for the Pearson correlation coefficients and deviations (AUEs) of the Δp*K*_a_ between methods (Figure S10); experimental conditions and p*K*_a_ values (Table S1); and experimental conditions and thermostability values (Table S2) (PDF)
