# Supplementary material for: Accurately Predicting Protein pKa Values Using Nonequilibrium Alchemy
Source: J Chem Theory Comput. 2023 Oct 11;19(21):7833–45. doi: 10.1021/acs.jctc.3c00721 (PMC10653114; doi:10.1021/acs.jctc.3c00721)
Supplement: Supplementary file 1 — ct3c00721_si_001.pdf [file ct3c00721_si_001.pdf]

Supporting Information: Accurately Predicting Protein pK_a Values Using Nonequilibrium Alchemy

Carter J. Wilson,[†,‡] Mikko Karttunen,[¶,§,‡] Bert L. de Groot,[‖] and Vytautas Gapsys[*,‖,⊥]

†*Department of Mathematics, The University of Western Ontario, N6A 5B7, London, Canada*
‡*Centre for Advanced Materials and Biomaterials Research (CAMBR), The University of Western Ontario, N6A 5B7, London, Canada*
¶*Department of Physics & Astronomy, The University of Western Ontario, N6A 5B7, London, Canada*
§*Department of Chemistry, The University of Western Ontario, N6A 5B7, London, Canada*
‖*Computational Biomolecular Dynamics Group, Department of Theoretical and Computational Biophysics, Max Planck Institute for Multidisciplinary Sciences, 37077 Göttingen, Germany*
⊥*Computational Chemistry, Janssen Research & Development, Janssen Pharmaceutica N. V., Turnhoutseweg 30, B-2340 Beerse, Belgium.*

E-mail: vgapsys@gwdg.de

Supplemental Methods

A crystallographic apo structure of human calmodulin is not available in the literature. We rebuilt human apo calmodulin using the apo structure of yeast calmodulin (PDB: 1LKJ[1]) and the AlphaFold2[2,3] structure of human calmodulin (AF-P0DP23-F1). The resultant structure had an unstructured linker similar to 1LKJ.

Supplemental Figures

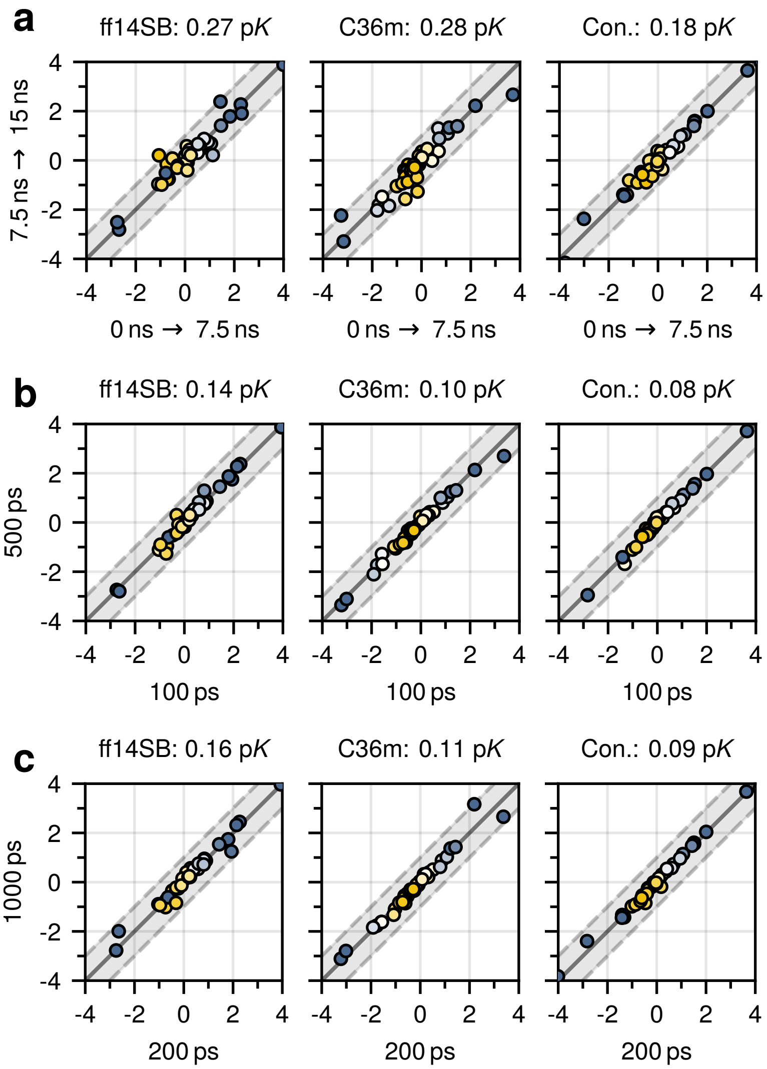

Figure S1: Convergence of transition morph generation and sampling scheme. Amber14SB and CHARMM36m forcefields (and consensus) are compared. Points represent the AUE to experiment and p*K* units in the title represent unsigned pK_a deviation between the two morph sampling strategies. **a.** Equilibration: initial morph structures extracted from the first half of a 15 ns (20 ns minus 5 ns equilibration) simulation versus those taken from the second half (marker color indicates AUE to experiment). **b.** Frequency of morph sampling: 100 ps vs 500 ps (i.e., 5 times the number of morphs). **c.** Morph transition time: 200 ps vs 1000 ps

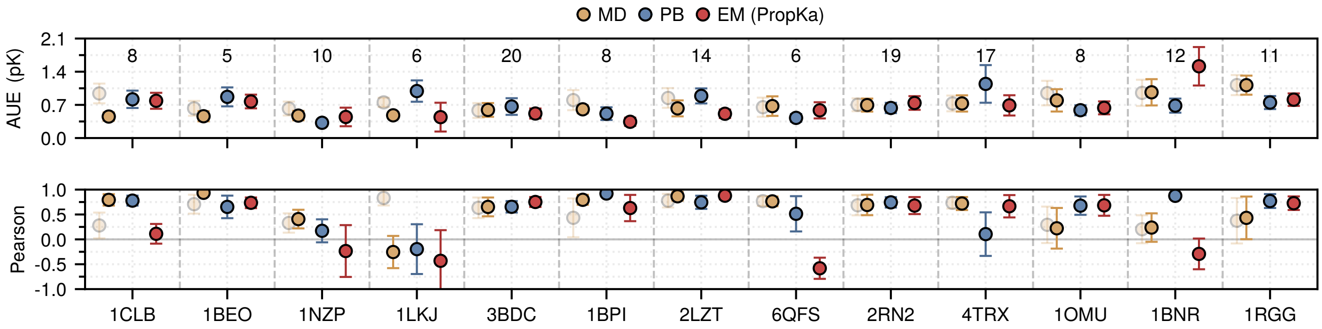

Figure S2: Protein-wise average unsigned errors (AUE) and Pearson correlation coefficients computed for the full dataset. MD-, PB-, and EM-based approaches are compared. For the MD approach both the adjusted and unadjusted (transparent marker) values are indicated (see the main text). Numerical values indicate the number of residues considered. Bootstrapped standard errors are depicted.

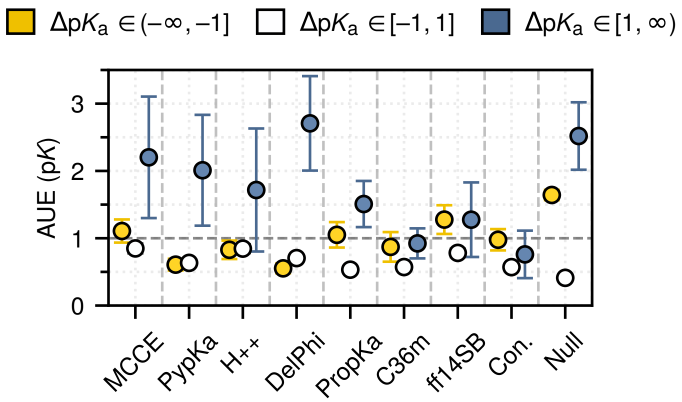

Figure S3: The average unsigned error of each method and a null model was assessed over the full dataset as a function of the ΔpK_a. Bootstrapped standard errors are depicted.

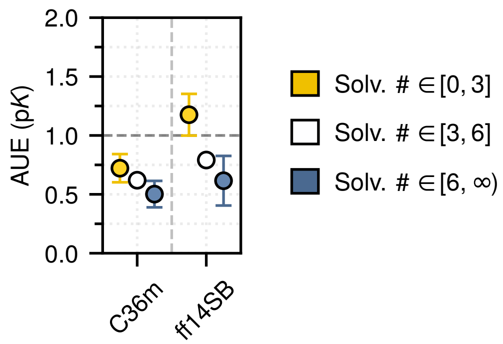

Figure S4: The average unsigned error of CHARMM36m and Amber14SB over the full dataset was assessed as a function of solvation number (computed from the radial distribution function of the center of mass of water and the carbonyl oxygens or lysine nitrogen). Bootstrapped standard errors are depicted.

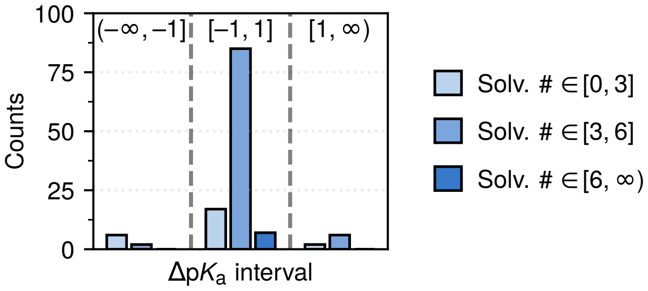

Figure S5: The average solvation number from CHARMM36m and Amber14SB was computed for residues as a function of the ΔpK_a interval.

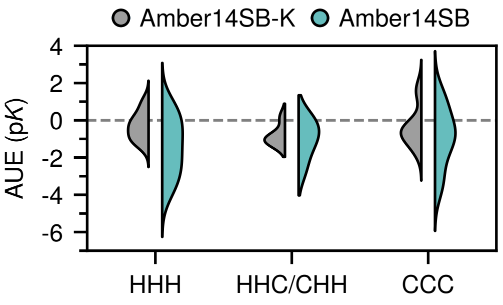

Figure S6: Distribution of the AUEs computed using the adjusted (Amber14SB-K) and unadjusted (Amber14SB) Amber force fields. Lysine residues were classified based on their secondary structure position as assigned by DSSP. HHH and CCC correspond to helical lysines flanked by helical residues and coiled lysines flanked by coiled residues, respectively. HHC/CHH correspond to lysine residues at the ends of helices.

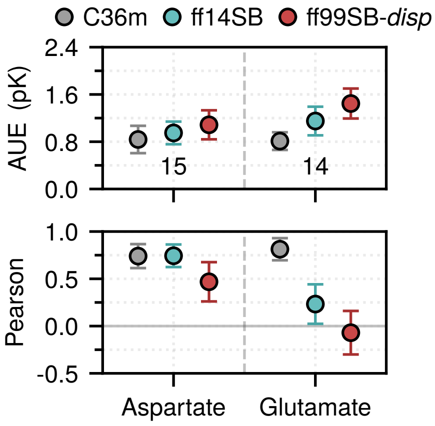

Figure S7: Residue-wise average unsigned errors (AUE) and Pearson correlation coefficients computed for a 29 residue subset. The performance of three force fields: CHARMM36m, Amber14SB, and Amber99SB-*disp*, was assessed. Numerical values (i.e., 15 and 14) indicate the number of residues considered. Bootstrapped standard errors are depicted.

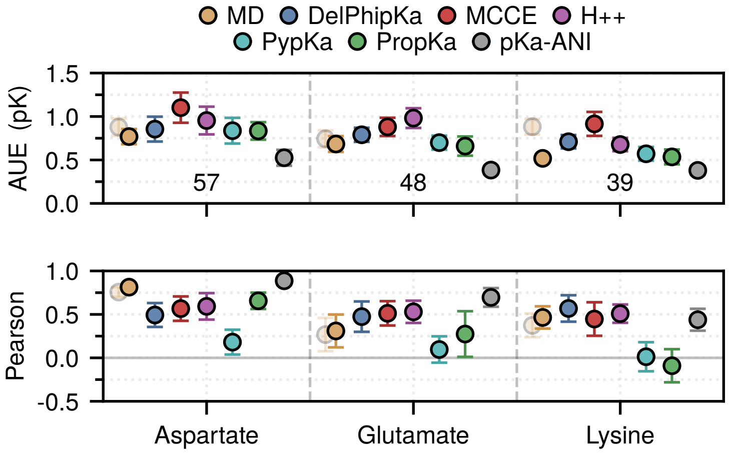

Figure S8: Residue-wise average unsigned errors (AUE) and Pearson correlation coefficients computed for the full dataset. A consensus force field NEQ approach (MD) in addition to six conventional predictor methods are compared. In the case of NEQ both the adjusted and unadjusted (transparent marker) values are indicated (see the main text). Numerical values indicate the number of residues considered. Bootstrapped standard errors are depicted.

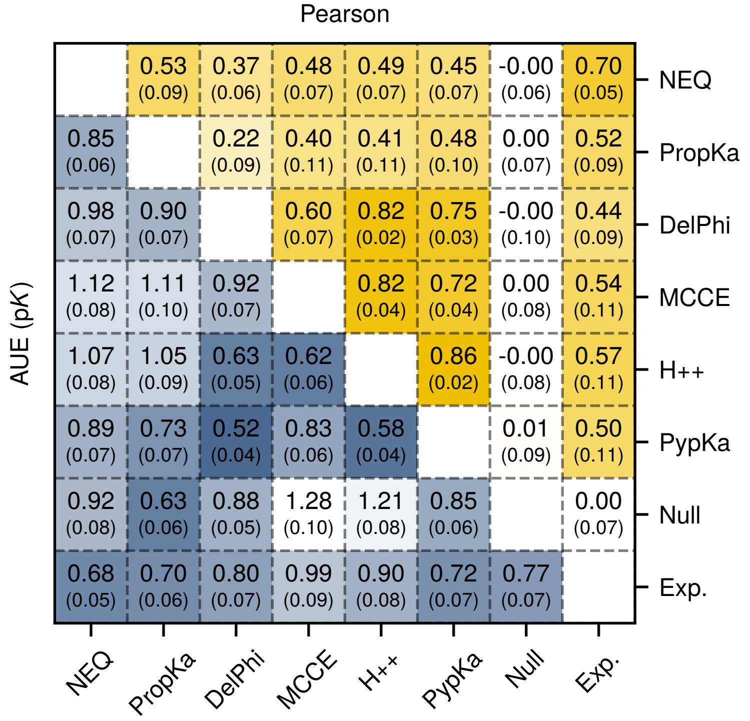

	NEQ	PropKa	DelPhi	MCCE	H++	PypKa	Null	Exp.
NEQ		0.53 (0.09)	0.37 (0.06)	0.48 (0.07)	0.49 (0.07)	0.45 (0.07)	-0.00 (0.06)	0.70 (0.05)
PropKa	0.85 (0.06)		0.22 (0.09)	0.40 (0.11)	0.41 (0.11)	0.48 (0.10)	0.00 (0.07)	0.52 (0.09)
DelPhi	0.98 (0.07)	0.90 (0.07)		0.60 (0.07)	0.82 (0.02)	0.75 (0.03)	-0.00 (0.10)	0.44 (0.09)
MCCE	1.12 (0.08)	1.11 (0.10)	0.92 (0.07)		0.82 (0.04)	0.72 (0.04)	0.00 (0.08)	0.54 (0.11)
H++	1.07 (0.08)	1.05 (0.09)	0.63 (0.05)	0.62 (0.06)		0.86 (0.02)	-0.00 (0.08)	0.57 (0.11)
PypKa	0.89 (0.07)	0.73 (0.07)	0.52 (0.04)	0.83 (0.06)	0.58 (0.04)		0.01 (0.09)	0.50 (0.11)
Null	0.92 (0.08)	0.63 (0.06)	0.88 (0.05)	1.28 (0.10)	1.21 (0.08)	0.85 (0.06)		0.00 (0.07)
Exp.	0.68 (0.05)	0.70 (0.06)	0.80 (0.07)	0.99 (0.09)	0.90 (0.08)	0.72 (0.07)	0.77 (0.07)	

Figure S9: Pearson correlations and AUE between ΔpK_a estimates were calculated for each method over the full dataset. Comparison with experiment means that the bottom row and rightmost column correspond to overall performance. Bootstrapped standard errors are indicated.

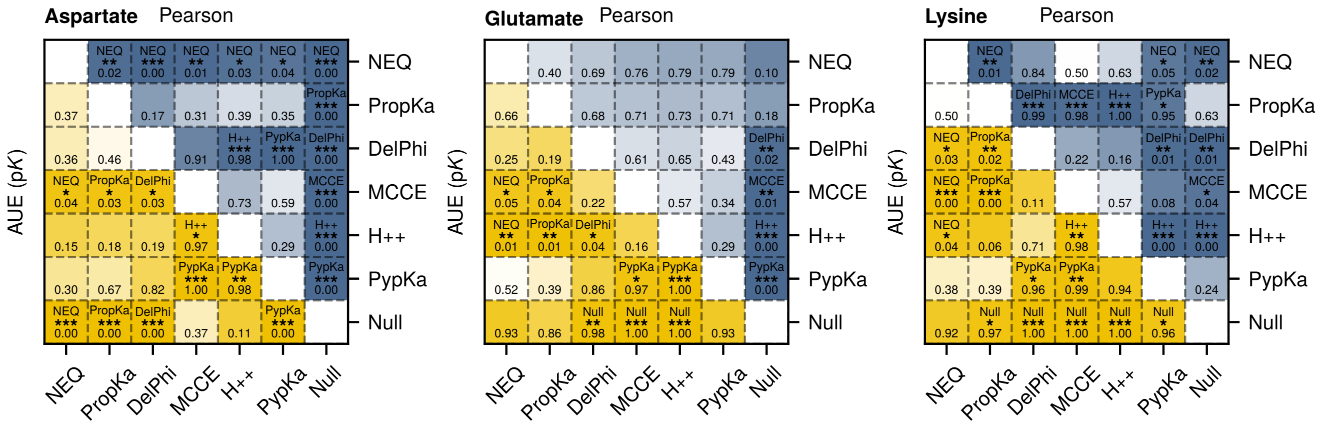

Figure S10: p-value analysis performed for each method and residue type over the full dataset. Yellow cells correspond to the question: is the method listed along the bottom different from the method listed on the right side with respect to the average unsigned error? Blue cells correspond to the question: is the method listed along the right side different from the method listed on the bottom with respect to the Pearson correlation coefficient? Asterisks indicate significance under a two-tailed statistical test[4] (i.e., [***] = $p \leq 0.005$, [**] = $p \leq 0.025$, and [*] = $p \leq 0.05$). The number in the cell indicates the area under the probability difference curve between the compared methods: ≤ 0.05 indicates the method is significantly better, while ≥ 0.95 indicates the method is significantly worse. The name of the method listed in the cell indicates which of the compared methods is better.

Table S1: pK_a datatable

ID	Temp.	Conc.	Cation	Anion	Residue	Exp. pK_a
1BPI	308	0.0	Na^+	Cl^-	D3	3.25 ± 0.15
1BPI	308	0.0	Na^+	Cl^-	E7	3.65 ± 0.05
1BPI	308	0.0	Na^+	Cl^-	K15	10.60
1BPI	308	0.0	Na^+	Cl^-	K26	10.60
1BPI	308	0.0	Na^+	Cl^-	K41	11.20
1BPI	308	0.0	Na^+	Cl^-	K46	10.30
1BPI	308	0.0	Na^+	Cl^-	E49	3.75 ± 0.05
1BPI	308	0.0	Na^+	Cl^-	D50	3.20 ± 0.20
1BNR	298	0.0	Na^+	Cl^-	D22	3.3 ± 0.1
1BNR	298	0.0	Na^+	Cl^-	D44	3.6 ± 0.1
1BNR	298	0.0	Na^+	Cl^-	E29	3.75 ± 0.05
1BNR	298	0.0	Na^+	Cl^-	E60	3.4 ± 0.1
1BNR	298	0.0	Na^+	Cl^-	D8	3.1 ± 0.2
1BNR	298	0.0	Na^+	Cl^-	D12	3.6 ± 0.2
1BNR	298	0.0	Na^+	Cl^-	D54	2.2 ± 0.3
1BNR	298	0.0	Na^+	Cl^-	D86	4.2 ± 0.1
1BNR	298	0.0	Na^+	Cl^-	D75	3.1 ± 0.2
1BNR	298	0.0	Na^+	Cl^-	E73	2.1 ± 0.1
1BNR	298	0.0	Na^+	Cl^-	D101	2.0 ± 0.2
1BNR	298	0.0	Na^+	Cl^-	D93	<2.0
1BEO	313	0.0	Na^+	Cl^-	D21	2.49 ± 0.05
1BEO	313	0.0	Na^+	Cl^-	D30	2.51 ± 0.09
1BEO	313	0.0	Na^+	Cl^-	K61	10.1
1BEO	313	0.0	Na^+	Cl^-	D72	2.61 ± 0.15
1BEO	313	0.0	Na^+	Cl^-	K94	9.4
3BDC	298	0.10	K^+	Cl^-	D19	2.21 ± 0.07
3BDC	298	0.10	K^+	Cl^-	D21	6.53 ± 0.02
3BDC	298	0.10	K^+	Cl^-	D40	3.87 ± 0.09
3BDC	298	0.10	K^+	Cl^-	D77	<2.2
3BDC	298	0.10	K^+	Cl^-	D83	<2.2
3BDC	298	0.10	K^+	Cl^-	D95	2.16 ± 0.07
3BDC	298	0.10	K^+	Cl^-	D143	3.8 ± 0.1
3BDC	298	0.10	K^+	Cl^-	D146	3.86 ± 0.05
3BDC	298	0.10	K^+	Cl^-	E10	2.82 ± 0.09
3BDC	298	0.10	K^+	Cl^-	E43	4.32 ± 0.04
3BDC	298	0.10	K^+	Cl^-	E52	3.93 ± 0.08
3BDC	298	0.10	K^+	Cl^-	E57	3.49 ± 0.09
3BDC	298	0.10	K^+	Cl^-	E67	3.76 ± 0.07
3BDC	298	0.10	K^+	Cl^-	E73	3.31 ± 0.01
3BDC	298	0.10	K^+	Cl^-	E75	3.26 ± 0.05
3BDC	298	0.10	K^+	Cl^-	E101	3.81 ± 0.1
3BDC	298	0.10	K^+	Cl^-	E122	3.89 ± 0.09
3BDC	298	0.10	K^+	Cl^-	E129	3.75 ± 0.09
3BDC	298	0.10	K^+	Cl^-	E135	3.76 ± 0.08
3BDC	298	0.10	K^+	Cl^-	E142	4.49 ± 0.04
1CLB	300	0.00	Na^+	Cl^-	K7	12.53
1CLB	300	0.00	Na^+	Cl^-	K12	12.02
1CLB	300	0.00	Na^+	Cl^-	K16	11.90
1CLB	300	0.00	Na^+	Cl^-	K25	13.21
1CLB	300	0.00	Na^+	Cl^-	K29	11.81
1CLB	300	0.00	Na^+	Cl^-	K41	11.02
1CLB	300	0.00	Na^+	Cl^-	K55	12.95
1CLB	300	0.00	Na^+	Cl^-	K71	11.22
1CLB	300	0.00	Na^+	Cl^-	K72	11.95

Table S1: pK_a datatable continued 1. An asterisk (*) indicates an ambiguous experimental assignment. In these cases the assignment that minimized the AUE for each individual predictor was used.

ID	Temp.	Conc.	Cation	Anion	Residue	Exp. pK_a
1RGG	295	0.10	Na^+	Cl^-	D17	3.72 ± 0.07
1RGG	295	0.10	Na^+	Cl^-	D25	4.87 ± 0.07
1RGG	295	0.10	Na^+	Cl^-	D33	2.39 ± 0.07
1RGG	295	0.10	Na^+	Cl^-	D79	7.37 ± 0.07
1RGG	295	0.10	Na^+	Cl^-	D84	3.01 ± 0.07
1RGG	295	0.10	Na^+	Cl^-	D93	3.09 ± 0.07
1RGG	295	0.10	Na^+	Cl^-	E14	5.02 ± 0.07
1RGG	295	0.10	Na^+	Cl^-	E41	4.14 ± 0.07
1RGG	295	0.10	Na^+	Cl^-	E54	3.42 ± 0.07
1RGG	295	0.10	Na^+	Cl^-	E74	3.47 ± 0.07
1RGG	295	0.10	Na^+	Cl^-	E78	3.13 ± 0.07
2LZT	300	0.10	Na^+	Cl^-	E7	2.85 ± 0.25
2LZT	300	0.10	Na^+	Cl^-	K13	10.50 ± 0.10
2LZT	300	0.10	Na^+	Cl^-	D18	2.66 ± 0.08
2LZT	300	0.10	Na^+	Cl^-	K33	10.60 ± 0.10
2LZT	300	0.10	Na^+	Cl^-	E35	6.20 ± 0.10
2LZT	300	0.10	Na^+	Cl^-	D48	1.60 ± 0.4
2LZT	300	0.10	Na^+	Cl^-	D52	3.68 ± 0.08
2LZT	300	0.10	Na^+	Cl^-	D66	0.90 ± 0.5
2LZT	300	0.10	Na^+	Cl^-	D87	2.07 ± 0.15
2LZT	300	0.10	Na^+	Cl^-	K96	10.80 ± 0.10
2LZT	300	0.10	Na^+	Cl^-	K97	10.30 ± 0.10
2LZT	300	0.10	Na^+	Cl^-	D101	4.09 ± 0.07
2LZT	300	0.10	Na^+	Cl^-	K116	10.40 ± 0.10
2LZT	300	0.10	Na^+	Cl^-	D119	3.2 ± 0.09
4TRX	298	0.00	Na^+	Cl^-	E6	4.8 ± 0.08
4TRX	298	0.00	Na^+	Cl^-	E13	4.4 ± 0.07
4TRX	298	0.00	Na^+	Cl^-	D16	4.1 ± 0.10
4TRX	298	0.00	Na^+	Cl^-	D20	3.8 ± 0.05
4TRX	298	0.00	Na^+	Cl^-	D26	9.9 ± 0.10
4TRX	298	0.00	Na^+	Cl^-	E47	4.1 ± 0.10
4TRX	298	0.00	Na^+	Cl^-	E56	3.1 ± 0.10
4TRX	298	0.00	Na^+	Cl^-	D58*	2.8 ± 0.10
4TRX	298	0.00	Na^+	Cl^-	D60*	4.2 ± 0.20
4TRX	298	0.00	Na^+	Cl^-	D61*	5.3 ± 0.30
4TRX	298	0.00	Na^+	Cl^-	D64	3.2 ± 0.06
4TRX	298	0.00	Na^+	Cl^-	E68	4.9 ± 0.07
4TRX	298	0.00	Na^+	Cl^-	E70	4.6 ± 0.08
4TRX	298	0.00	Na^+	Cl^-	E88	3.7 ± 0.08
4TRX	298	0.00	Na^+	Cl^-	E95	4.1 ± 0.02
4TRX	298	0.00	Na^+	Cl^-	E98	3.9 ± 0.02
4TRX	298	0.00	Na^+	Cl^-	E103	4.4 ± 0.07
2RN2	300	0.10	Na^+	Cl^-	E6	4.5
2RN2	300	0.10	Na^+	Cl^-	D10	6.1
2RN2	300	0.10	Na^+	Cl^-	E32	3.6
2RN2	300	0.10	Na^+	Cl^-	E48	4.4
2RN2	300	0.10	Na^+	Cl^-	E57	3.2
2RN2	300	0.10	Na^+	Cl^-	E61	3.9
2RN2	300	0.10	Na^+	Cl^-	E64	4.4
2RN2	300	0.10	Na^+	Cl^-	D70	2.6
2RN2	300	0.10	Na^+	Cl^-	D94	3.2
2RN2	300	0.10	Na^+	Cl^-	D108	3.2
2RN2	300	0.10	Na^+	Cl^-	D134	4.1
2RN2	300	0.10	Na^+	Cl^-	E119	4.1

Table S1: pK_a datatable continued 2

ID	Temp.	Conc.	Cation	Anion	Residue	Exp. pK_a
2RN2	300	0.10	Na^+	Cl^-	E129	3.6
2RN2	300	0.10	Na^+	Cl^-	E131	4.3
2RN2	300	0.10	Na^+	Cl^-	E135	4.3
2RN2	300	0.10	Na^+	Cl^-	E147	4.2
2RN2	300	0.10	Na^+	Cl^-	E154	4.4
1OMU	298	0.015	K^+	Cl^-	D7	2.95 ± 0.03
1OMU	298	0.015	K^+	Cl^-	E10	2.15 ± 0.03
1OMU	298	0.015	K^+	Cl^-	D27	2.56 ± 0.03
1OMU	298	0.015	K^+	Cl^-	E43	2.41 ± 0.03
1OMU	298	0.015	K^+	Cl^-	K13	9.86 ± 0.03
1OMU	298	0.015	K^+	Cl^-	K29	11.12 ± 0.02
1OMU	298	0.015	K^+	Cl^-	K34	10.13 ± 0.05
1OMU	298	0.015	K^+	Cl^-	K55	11.10 ± 0.06
6QFS (T39D)	298	0.10	K^+	Cl^-	D39	5
6QFS (T39D,T66H)	298	0.10	K^+	Cl^-	D39	4.07 ± 0.02
6QFS (S43D)	298	0.10	K^+	Cl^-	D43	4.37 ± 0.01
6QFS (S43D,T66H)	298	0.10	K^+	Cl^-	D43	4.32 ± 0.02
6QFS (S61D)	298	0.10	K^+	Cl^-	D61	4.15 ± 0.01
6QFS (S61D,T66H)	298	0.10	K^+	Cl^-	D61	4.10 ± 0.02
1NZP	298	0.10	K^+	Cl^-	K8	10.27 ± 0.14
1NZP	298	0.10	K^+	Cl^-	K19	10.07 ± 0.14
1NZP	298	0.10	K^+	Cl^-	K25	10.23 ± 0.14
1NZP	298	0.10	K^+	Cl^-	K33	10.07 ± 0.14
1NZP	298	0.10	K^+	Cl^-	K41	10.01 ± 0.14
1NZP	298	0.10	K^+	Cl^-	K47	10.65 ± 0.14
1NZP	298	0.10	K^+	Cl^-	K51	10.26 ± 0.14
1NZP	298	0.10	K^+	Cl^-	K67	10.16 ± 0.14
1NZP	298	0.10	K^+	Cl^-	K72	9.58 ± 0.14
1NZP	298	0.10	K^+	Cl^-	K84	10.27 ± 0.14
1LKJ	298	0.10	K^+	Cl^-	K13	10.23 ± 0.10
1LKJ	298	0.10	K^+	Cl^-	K21	10.55 ± 0.10
1LKJ	298	0.10	K^+	Cl^-	K30	10.55 ± 0.10
1LKJ	298	0.10	K^+	Cl^-	K75	9.87 ± 0.10
1LKJ	298	0.10	K^+	Cl^-	K77	10.55 ± 0.10
1LKJ	298	0.10	K^+	Cl^-	K94	10.55 ± 0.10

Table S2: Thermostability datatable

ID	Temp.	Conc.	Cation	Anion	Mutation	Exp. ΔΔG (kcal/mol)
1EY0	293	0.00	K^+	CL^-	V66K	–7.5
1EY0	293	0.00	K^+	CL^-	K116G	1.0
2LZM	298	0.20	K^+	CL^-	K43A	–1.0
2LZM	298	0.20	K^+	CL^-	S44K	0.2
2LZM	298	0.025	K^+	CL^-	M106K	–3.4
2LZM	298	0.025	K^+	CL^-	M120K	–1.6
2LZM	298	0.025	K^+	CL^-	K16E	0.5
2LZM	298	0.10	NA^+	CL^-	K85A	–0.6
2LZM	298	0.10	NA^+	CL^-	R96K	0.0
2LZM	298	0.20	K^+	CL^-	K124G	–0.1
2LZM	298	0.025	K^+	CL^-	K135E	–1.0
2LZM	298	0.025	K^+	CL^-	K147E	–0.7
2RN2	298	0.00	K^+	CL^-	K95A	0.1
2RN2	298	0.00	K^+	CL^-	K95G	1.9
2RN2	298	0.00	K^+	CL^-	K95N	0.9

References

(1) Ishida, H.; ichi Nakashima, K.; Kumaki, Y.; Nakata, M.; Hikichi, K.; Yazawa, M. The Solution Structure of Apocalmodulin from Saccharomyces cerevisiae Implies a Mechanism for Its Unique Ca2+ Binding Property. *Biochemistry* **2002**, *41*, 15536–15542.

(2) Jumper, J.; Evans, R.; Pritzel, A.; Green, T.; Figurnov, M.; Ronneberger, O.; Tunyasuvunakool, K.; Bates, R.; Žídek, A.; Potapenko, A.; Bridgland, A.; Meyer, C.; Kohl, S. A. A.; Ballard, A. J.; Cowie, A.; Romera-Paredes, B.; Nikolov, S.; Jain, R.; Adler, J.; Back, T.; Petersen, S.; Reiman, D.; Clancy, E.; Zielinski, M.; Steinegger, M.; Pacholska, M.; Berghammer, T.; Bodenstein, S.; Silver, D.; Vinyals, O.; Senior, A. W.; Kavukcuoglu, K.; Kohli, P.; Hassabis, D. Highly accurate protein structure prediction with AlphaFold. *Nature* **2021**, *596*, 583–589.

(3) Varadi, M.; Anyango, S.; Deshpande, M.; Nair, S.; Natassia, C.; Yordanova, G.; Yuan, D.; Stroe, O.; Wood, G.; Laydon, A.; Žídek, A.; Green, T.; Tunyasuvunakool, K.; Petersen, S.; Jumper, J.; Clancy, E.; Green, R.; Vora, A.; Lutfi, M.; Figurnov, M.; Cowie, A.; Hobbs, N.; Kohli, P.; Kleywegt, G.; Birney, E.; Hassabis, D.; Velankar, S. AlphaFold Protein Structure Database: massively expanding the structural coverage of protein-sequence space with high-accuracy models. *Nucleic Acids Res.* **2021**, *50*, D439–D444.

(4) Hahn, D.; Bayly, C.; Boby, M. L.; Bruce Macdonald, H.; Chodera, J.; Gapsys, V.; Mey, A.; Mobley, D.; Perez Benito, L.; Schindler, C.; Tresadern, G.; Warren, G. Best Practices for Constructing, Preparing, and Evaluating Protein-Ligand Binding Affinity Benchmarks. *LiveCoMS* **2022**, 1497.
